# Explore prognostic biomarker of bladder cancer based on competing endogenous network

**DOI:** 10.1042/BSR20202463

**Published:** 2020-12-02

**Authors:** Faping Li, Hui Guo, Bin Liu, Nian Liu, Zhixiang Xu, Yishu Wang, Honglan Zhou

**Affiliations:** 1 Department of Urology, the First Hospital of Jilin University, Changchun 130021, Jilin, China; 2 Key Laboratory of Pathobiology, Ministry of Education, Jilin University, Changchun 130021, Jilin, China

**Keywords:** Bladder cancer, ceRNA, lncRNA, Overall survival, Prognosis

## Abstract

Bladder cancer (BC) is the most common tumor of the urinary tract. Increasing evidence showed that long non-coding RNA (lncRNA) is a critical regulator in cancer development and progression. However, the functions of lncRNAs in the development of BC remain mostly undefined. In the present study, based on RNA sequence profiles from The Cancer Genome Atlas database, we identified 723 lncRNAs, 157 miRNAs, and 1816 mRNAs aberrantly expressed in BC tissues. A competing endogenous RNA network, including 49 lncRNAs, 17 miRNAs, and 36 mRNAs, was then established. The functional enrichment analyses showed that the mRNAs in the ceRNA network mainly participated in ‘regulation of transcription’ and ‘pathways in cancer’. Moreover, the Cox regression analyses demonstrated that three lncRNAs (AC112721.1, TMPRSS11GP, and ADAMTS9-AS1) could serve as independent risk factors. We established a risk prediction model with these lncRNAs. Kaplan–Meier curve analysis showed that high-risk patients’ prognosis was lower than that of low-risk patients (*P*=0.001). The present study provides novel insights into the lncRNA-mediated ceRNA network and the potential of lncRNAs to be candidate prognostic biomarkers in BC, which could help better understand the pathological changes and pathogenesis of BC and be useful for clinical studies in the future.

## Introduction

Bladder cancer (BC) is the most common neoplasm in the urinary system, which has a high recurrence and mortality rate due to occult onset and lack of effective detection methods [1,2]. Even with advanced medical technology, the therapeutic effect and prognosis of patients with BC are still less than desirable [3,4]. Thus, it is necessary to identify novel candidate prognostic biomarkers and therapeutic targets for treating BC effectively.

Long non-coding RNA (lncRNA) is a subtype of ncRNA, which is not involved in protein-coding directly [5,6]. Increasing evidence has confirmed that lncRNAs play significant roles in regulating chromatin organization, transcription, nuclear domains, and messenger RNA (mRNA) stability and translation [7,8]. It has been reported that lncRNAs are eligible to be diagnostic or prognostic biomarkers of multiple types of cancer, including pancreatic cancer [9], renal cancer [10], and gastric cancer [11]. Even now, certain lncRNAs associated with the prognosis of BC have come under observation [12–14], valid lncRNAs serving as predictive biomarkers of this disease remain tiny.

In 2011, the competing endogenous RNA (ceRNA) hypothesis was put forward for the first time in the paper published in Cell by Salmena [15]. This hypothesis provided a novel ‘RNA language’ in which microRNA (miRNA) response elements (MREs) were appointed as the letters to make RNAs ‘talk’ with each other [15]. LncRNAs containing the same MREs as mRNA may function as ceRNAs and regulate mRNA translation by competing for shared miRNAs [16,17]. For example, a study has demonstrated that lncRNA MATN1-AS1 promotes glioma progression through sponging miR-200b/c/429 to mediate CHD1 expression [18]. Collectively, these studies indicate that abnormal expression of lncRNAs in the ceRNA network may result in cancer initiation and progression. Nevertheless, fewer studies have been reported on the ceRNA network in BC.

**Figure 1:**
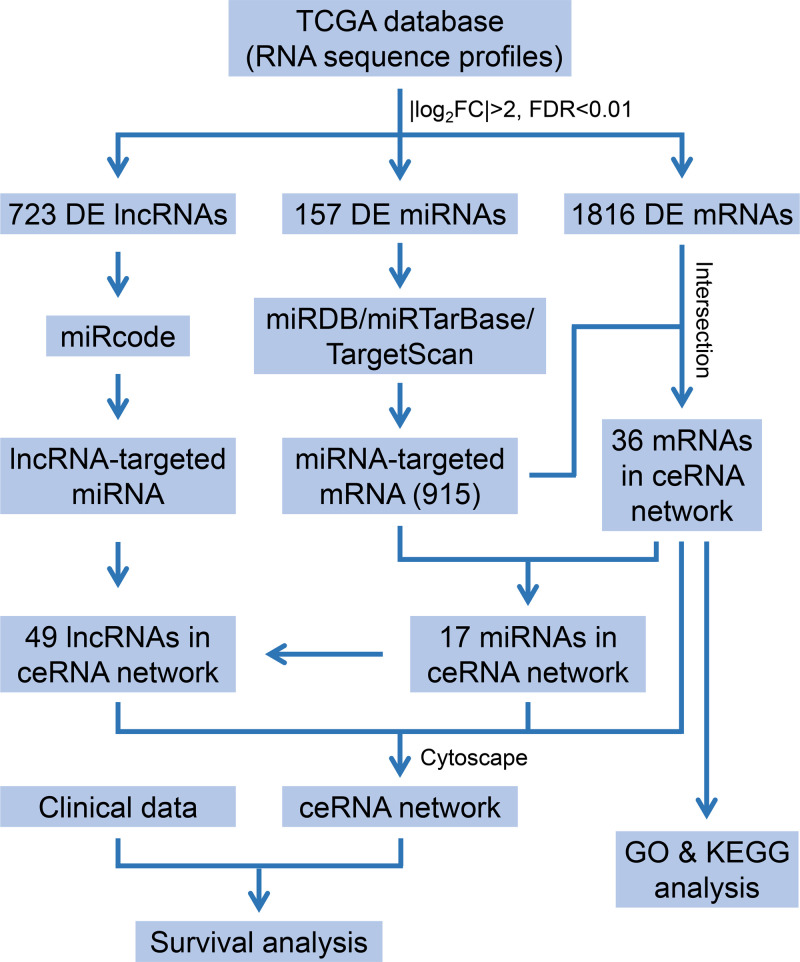
Flow chart of the construction of the ceRNA network and bioinformatics analysis in bladder cancer

In the present research, we downloaded significant RNA sequence profiles and relevant clinical data from The Cancer Genome Atlas (TCGA) database (https://portal.gdc.cancer.gov/). After screening and comparing, we constructed an lncRNA-mediated ceRNA network of BC. The functional enrichment analysis and survival analysis were then carried out to investigate the potential mechanism and prognostic biomarkers of BC. Subsequently, via a comprehensive bioinformatic analysis, we identified a novel three-lncRNA signature that might be useful as a potential independent prognostic factor for BC. A flowchart was provided as a general description of how the study was carried out (Figure 1).

## Materials and methods

### Data acquisition from TCGA database

RNA sequence profiles and clinical data of BC cases were downloaded from the TCGA database. The Genomic Data Commons Transfer Tool was used to obtain the gene expression profiles. The inclusion criteria were: (i) expression data available, (ii) overall survival time for more than 30 days. No approval is needed from the ethics committee because all the information required from the TCGA database is open access and public.

### Identification of differentially expressed RNAs

The acquired RNA data matrices were pre-processed and normalized. Then, the ‘edgeR’ package in R (version 3.6.1) was used for differential expression analysis between the BC and adjacent-normal bladder tissues. False discovery rate (FDR) < 0.01 and log fold change (log_2_FC) > 2 or < -2 were considered statistically significant. The heat maps and volcano plots of differentially expressed RNAs (DE RNAs) were visualized using the ‘ggplot2’ and ‘pheatmap’ packages in R.

### Construction of a ceRNA network

Based on the ceRNA hypothesis, a ceRNA network, including lncRNA, miRNA, and mRNA, was constructed. Briefly, the interaction pairs of DE lncRNA with DE miRNA were predicted using the miRcode online software (http://www.mircode.org/). Then, the mRNAs targeted by miRNAs were predicted according to miRTarBase (http://mirtarbase.mbc.nctu.edu.tw//), TargetScan (http://www.targetscan.org//), and miRDB (http://www.mirdb.org/). The obtained mRNAs were used to intersect with the DE mRNAs to identify final targeted mRNAs. Finally, the ceRNA network was constructed relying on the co-expression network of interactions among DE lncRNAs, DE miRNAs and DE mRNAs, and was visualized using Cytoscape (version 3.7.2).

### Functional enrichment analysis

The Gene Ontology (GO) is a freely available bioinformatics resource and widely used to offer information about gene product function [19]. Similarly, the Kyoto Encyclopedia of Genes and Genomes (KEGG) is a database resource that integrates genomic, chemical, and system functional information [20]. In the ceRNA network, lncRNAs regulate the activity of mRNAs indirectly through sponging miRNA. Therefore, we analyze the functional enrichment to explore the function of DE lncRNA in the network using the Database for Annotation, Visualization and Integrated Discovery (https://david.ncifcrf.gov/). GO and KEGG terms with *P*<0.05 were considered significant. The results were plotted using the ‘ggplot2’ R package.

### Construction of a risk score model and survival analysis

The univariate and multivariate Cox regression analyses were used to determine the independent prognostic biomarkers for BC. Kaplan–Meier curves visualized the relationship between the predictive lncRNAs and the overall survival of patients with BC. Besides, the risk scores were calculated using the equation below.

$$\text{Risk score }={\text{ Exp}}_{\text{lncRNA1}}\times \beta_{\text{lncRNA1}}+{\text{ Exp}}_{\text{lncRNA2}}\times \beta_{\text{lncRNA2}}+\;\cdots {\text{Exp}}_{\text{lncRNAn}}\times \beta_{\text{lncRNAn}}$$

(In equation, ‘Exp’ represents the expression level of lncRNAs. ‘β’ is the regression coefficient acquired from the multivariate Cox regression analysis.)

The median risk score was used as the threshold for the classification of patients with BC into high- and low-risk groups. The relationship between risk score and overall survival time was visualized by Kaplan–Meier curves and was evaluated with the log-rank test with a significance level of *P<*0.05. The receiver operating characteristic (ROC) curve was used to evaluate the predictive model's performance by calculating the area under the curve (AUC). In addition, the ROC curve was visualized using the ‘survival ROC’ package in R.

## Results

### Characteristics of clinical features in the TCGA database

Clinical information of BC patients from the TCGA database was available in 400 of 433 cases. The clinical characteristics including age, gender, tumor stage and grade, lymph node metastasis, and invasiveness of the BC patients are presented in Table 1. The median age of the patients was 68 years (range: 34–89 years). Significantly, all clinical features other than gender were markedly associated with survival. BC patients with upper age, high-grade, later stage, and lymphatic metastasis were associated with adverse outcomes.

**Table 1:** The clinicopathological characteristics of bladder cancer patients

Variables	Patients (%)	Dead	χ^2^	*P-*value
Age				
>60	293 (73.25)	145	12.182	<0.001
≤60	107 (26.75)	32		
Gender				
Female	105 (26.25)	51	1.078	0.299
Male	295 (73.75)	126		
Stage				
I & II	128 (32.00)	34	23.871	<0.001
III & IV	272 (68.00)	143		
Grade				
High	379 (94.75)	175	10.834	0.001
Low	21 (5.25)	2		
Lymph node				
Yes	128 (32.00)	82	33.412	<0.001
No	231 (57.75)	75		
Unknown	41 (10.25)	20		

### Identification of DE lncRNAs, DE mRNAs, and DE miRNAs

**Figure 2:**
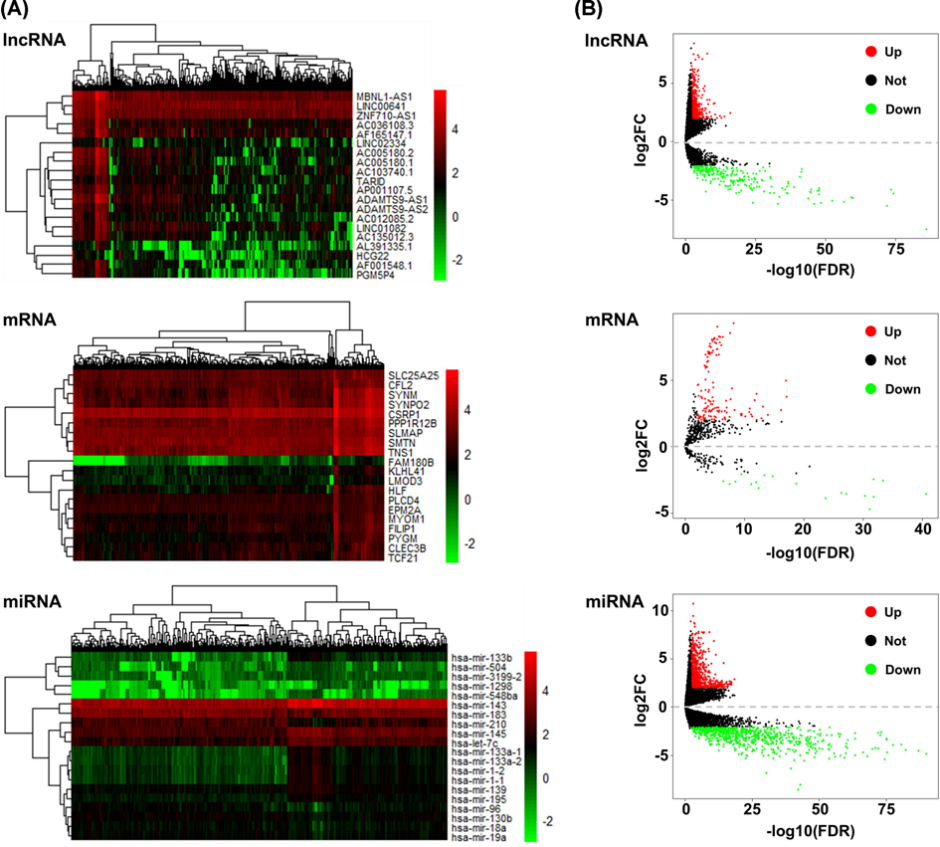
Differentially expressed RNAs of BC (**A**) Heat maps of the top 20 DE RNAs (lncRNA, miRNA, and mRNA) between BC and normal bladder tissues (FDR < 0.01, |log2FC| > 2). Red indicates high expression, and green indicates low expression. (**B**) Volcano plots of the DE lncRNAs, DE miRNAs, and DE mRNAs between BC and normal bladder tissues (FDR < 0.01, |log2FC| > 2). Red dots indicate up-regulated RNAs, green dots indicate down-regulated RNAs, and the black dots indicate no difference in expression.

The RNA sequence profiles of BC and adjacent-normal bladder tissues downloaded from the TCGA database were pre-processed to identify the DE lncRNAs, DE mRNAs, and DE miRNAs. A total of 723 DE lncRNAs (471 up- and 252 down-regulated), 1816 DE mRNAs (1005 up- and 811 down-regulated), and 157 DE miRNAs (136 up- and 21 down-regulated) were identified using the ‘edgeR’ package in R software, typically with FDR < 0.01 and |log_2_FC| > 2. The heat maps showed the expression profiles of the 20 top-ranking DE RNAs (Figure 2A). Volcano plots presented the distribution of DE RNAs were illustrated according to–log_10_FDR as abscissa and log_2_FC as coordinate (Figure 2B).

### Construction of the lncRNA-mediated ceRNA network

The lncRNA-mediated ceRNA network was constructed to investigate the mechanism by which lncRNAs influenced the expression of mRNAs in BC. According to the ceRNA hypothesis principle, lncRNAs might regulate gene expression by competitively binding microRNA through MREs. Therefore, we first evaluated the relationships between 723 DE lncRNAs and 157 DE miRNAs using the miRcode database. A total of 409 pairs of lncRNAs and miRNAs were then identified, which included 80 lncRNAs and 21 miRNAs. Based on the above 21 miRNAs, 915 target mRNAs were identified using the miRDB, miRTarBase, and TargetScan databases. Finally, there were 36 BC-specific mRNAs in the ceRNA network after intersecting target mRNAs with 1816 DE mRNA.

**Figure 3:**
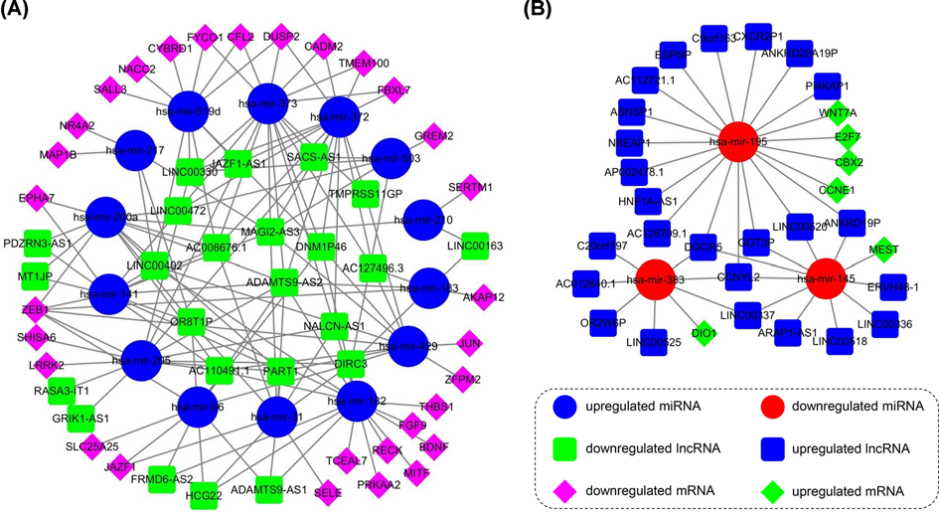
The ceRNA networks based on the expression levels of miRNA in BC (**A**) The up-regulated miRNAs-centric network shows the competitive relationship between down-regulated lncRNAs and mRNAs. (**B**) The down-regulated miRNAs-centric network shows the competitive relationship between up-regulated lncRNAs and mRNAs.

On the basis of the above data, the ceRNA network that consisted of 49 lncRNAs, 17 miRNAs, and 36 mRNAs was established and visualized using Cytoscape. As shown in Figure 3, two networks were constructed based on the expression levels of miRNA in BC.

### Functional enrichment analysis of DE mRNAs in the ceRNA network

**Figure 4:**
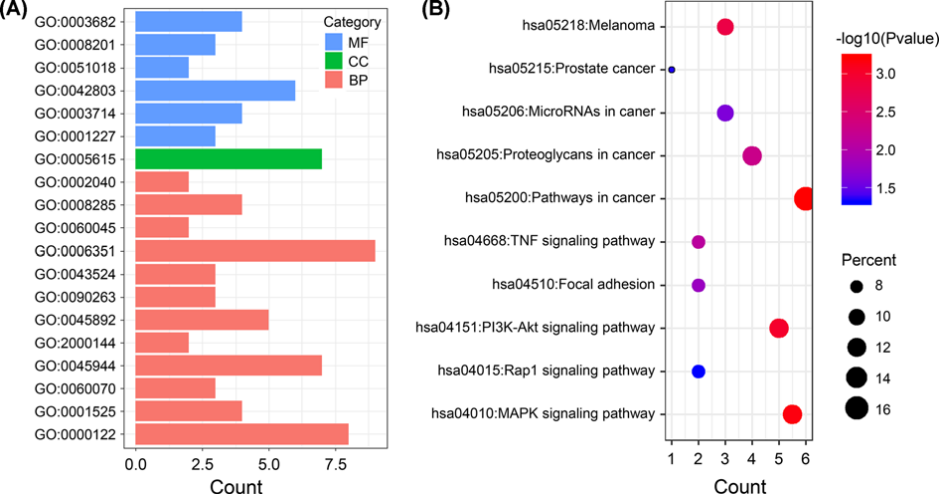
GO terms and KEGG pathways of DEGs significantly enriched in BC (**A**) Enriched GO biological process terms. The horizontal axis denotes the number of DE mRNAs enriched in GO terms. The vertical axis denotes different GO terms, including molecular function, cellular component, and biological process. (**B**) KEGG pathways analysis. The horizontal axis and vertical axis denote the number of DE mRNAs and different KEGG pathways, respectively.

To better understand the biological functions of DE lncRNAs in the ceRNA network, we analyzed the 36 mRNAs of the ceRNA network using GO and KEGG analyses. The results of the GO analysis indicated that the mRNAs were mainly enriched in the molecular function (MF) and biological process (BP) (Figure 4A). The mRNAs related to MF were most relevant to protein homodimerization activity, transcription corepressor activity, and chromatin binding. In terms of BP, mRNAs were mainly associated with DNA-templated transcription and negative regulation of transcription from RNA polymerase II promoter (Supplementary Table S1).

The results of KEGG analysis showed that four main enriched pathways were identified, including ‘MicroRNAs in cancer’, ‘Pathways in cancer’, ‘mitogen-activated protein kinase (MAPK) signaling pathway’, and ‘phosphatidylinositol-3 kinase-protein kinase B (PI3K-Akt) signaling pathway’ (Figure 4B and Supplementary Table S2). Dysregulation of miRNAs has been widely observed in different types of cancer via the degradation of target mRNAs [21,22]. Signaling pathways play a significant role in living organisms. Still, multiple signaling pathways have been identified as oncogenesis and cancer progression drivers, such as the ‘MAPK signaling pathway’ and ‘PI3K-Akt signaling pathway’ [23–25]. These results indicated that these lncRNA-mediated mRNAs were involved in the pathophysiology and development of BC.

### Construction of the risk score model and survival analysis

**Figure 5:**
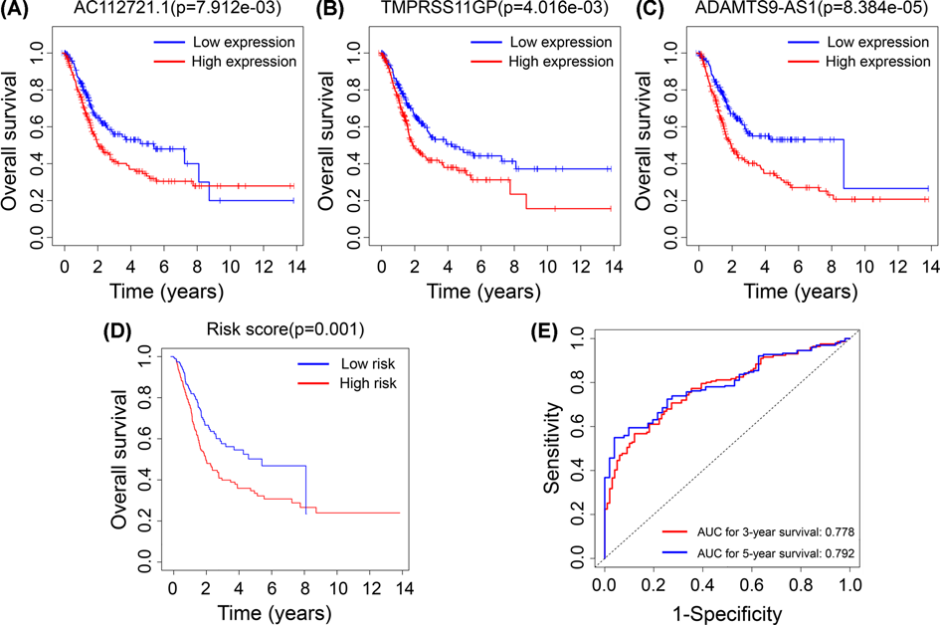
Correlations of the prognosis-associated lncRNAs and risk score with overall survival (**A–C**) Kaplan–Meier survival curves for patients grouped by expression of AC112721.1, TMPRSS11GP, and ADAMTS9-AS1. (**D**) Kaplan–Meier survival curves for patients with low- vs. high-risk scores. (**E**) ROC curves of the risk model for predicting 3- and 5-year survival rates.

Of 49 lncRNAs in the ceRNA network, ten lncRNAs associated with prognosis and overall survival were identified using univariate Cox regression analysis (Table 2). Subsequently, the ten lncRNAs were evaluated using multivariate Cox regression analysis. As a result, only three lncRNAs (AC112721.1, TMPRSS11GP, and ADAMTS9-AS1) had a significant independent prognostic value in BC with *P<*0.05 (Table 2 and Figure 5A–C). We further investigated the relationship between the three lncRNAs and clinicopathological characteristics of BC patients. The results showed that the expressions of AC112721.1 and ADAMTS9-AS1 were significantly associated with lymph node metastasis, tumor grade, and pathologic stage of BC (*P*<0.05), while the expression of TMPRSS11GP was significantly associated with age and tumor grade (*P*<0.05) (Supplementary Table S3). Further, the patients in the high expression group tended to have higher tumor stages and grades.

**Table 2:** Univariate and multivariate Cox regression analysis of prognostic factors for overall survival

Variables	Univariate analysis		Multivariate analysis	
	HR (95%CI)	*P* value	HR (95%CI)	*P* value
AC112721.1	1.148 (1.060–1.243)	6.57E-04	1.130 (1.027–1.243)	0.012
ADAMTS9-AS1	1.111 (1.043–1.184	0.001	1.140 (1.006–1.291)	0.040
TMPRSS11GP	1.123 (1.024–1.231)	0.014	1.119 (1.010–1.239)	0.031
ADAMTS9-AS2	1.114 (1.023–1.213)	0.013		
HCG22	1.122 (1.023–1.230)	0.015		
LINC00525	0.917 (0.850–0.989)	0.025		
FRMD6-AS2	1.125 (1.010–1.252)	0.032		
PDZRN3-AS1	1.189 (1.010–1.400)	0.038		
SACS-AS1	1.166 (1.004–1.354)	0.044		
AC110491.1	1.089 (1.000–1.185)	0.048		

Abbreviations: 95%CI, 95% confidence interval; HR, Hazard ratio.

In addition, the risk score model was constructed based on the expression level of the three lncRNAs outlined above. A total of 400 patients with BC were screened out and divided into high- and low-risk groups with the median risk score as the threshold. The Kaplan–Meier survival analysis revealed that the high-risk group’s overall survival was worse than that of the low-risk group (*P=*0.001, Figure 5D). The ROC curves were plotted to determine the optimal cutoff points for the three-lncRNA signature in predicting 3- and 5-year survival. The AUC values were 0.778 and 0.792 for 3- and 5-year survival, respectively, implying the moderate accuracy in prediction (Figure 5E).

## Discussion

BC is one of the most common malignant tumors worldwide with remarkable morbidity and mortality [26]. Although 70–80% of first diagnosed cases are non-muscle-invasive bladder cancer (NMIBC), more than a third of NMIBC will continue to progress and metastasize over time [27,28]. Hideously, muscle invasion and metastasis substantially reduce the 5-year survival rate and contribute to a high mortality rate for patients with BC [29]. Accordingly, it is imperative to find novel biomarkers for predicting and diagnosing BC accurately. Growing experimental evidence indicates that the abnormal expression of ncRNAs, including lncRNAs and miRNAs, is intimately relevant to malignant progression and metastasis of cancer [30–33].

Since the ceRNA hypothesis has been proposed, researchers are increasingly taking an interest in the ceRNA network in which lncRNAs may affect the transcription and expression of mRNA by interacting with miRNAs. For instance, Miao et al. [34] identified the DE lncRNAs between 13 BC and eight normal adjacent tissue samples via microarray analysis and investigated the biological functions of LINC00612 *in vitro* and *in vivo*. The results indicated that LINC00612 served as a ceRNA to up-regulate PHF14 expression and then promoted BC cell proliferation and invasion. Another study showed that lncRNA ZEB1-AS1 functioned as a ceRNA in BC to regulate the expression of protein-coding gene fascin-1 through miR-200b [35]. However, the accuracy of the above results is limited due to the few samples.

In the present study, based on data from the TCGA database, we analyzed expression profiles of three kinds of RNA of more than 400 BC tissues and 19 adjacent-normal tissues and then performed the ceRNA network analysis. As a result, 723 lncRNAs, 157 miRNAs, and 1816 mRNAs were found to be differentially expressed in BC with FDR < 0.01 and |log_2_FC| > 2 as the threshold, of which 49 lncRNAs, 17 miRNAs, and 36 mRNAs were identified to construct the ceRNA network. To explore the functions of lncRNAs in the ceRNA network, the enrichment of functions and signaling pathways of the 36 mRNAs were conducted using GO and KEGG analyses. The results showed that these mRNAs were mainly enriched in ‘nuclear domains’, indicating that lncRNAs acting as ceRNAs might play significant roles in regulating transcription, stability, and translation of mRNA.

In addition, we predicted that the lncRNA-mediated 36 mRNAs were related to the miRNAs and signaling pathways, such as the ‘MAPK signaling pathway’ and ‘PI3K-Akt signaling pathway’. It has been confirmed that abnormal expression of miRNA was related to oncogenesis. Cheng et al. [36] reported that the expression of miR-200c was significantly up-regulated in BC compared with the adjacent normal tissues and directly targeted the mRNA RECK to promote the cell migration and invasion of BC. MAPK signaling pathway is a reasonably complicated pathway associated with cell proliferation, invasion, metastasis, and survival processes. Kumar et al*.* [37] confirmed the facilitating effect of the MAPK pathway on cell proliferation, progression, and survival of BC through treating BC cells with MAPK specific inhibitors. Moreover, the PI3K-AKT signaling pathway is one of the most frequent activated pathways in various cancers, while its aberrant activation will reprogramme cellular metabolism for the sake of supporting survival and proliferation of cancer cells [25]. Lin et al*.* [38] reported that glaucocalyxin A induced apoptosis and G2/M cell cycle arrest of BC cells by inhibiting the PI3K-AKT signaling pathway, indicating that targeting PI3K-AKT signaling may be a potential therapeutic method for BC. The mechanism analysis related to the ceRNA network is meaningful and necessary to provide new insights into early detection and BC therapies.

To explore BC’s prognostic biomarker based on the ceRNA network, we investigated the association of lncRNAs in the ceRNA network and overall survival in BC patients using the univariate and multivariate Cox regression. The analyses showed that three lncRNAs (AC112721.1, TMPRSS11GP, and ADAMTS9-AS1) possessed significant independent prognostic value in BC, which was consistent with the previous result [39]. However, to the best of our knowledge, the present study is the first to report on the relationship between TMPRSS11GP expression and prognosis of BC patients. Previous studies also attempted to explore BC-related biomarkers through the construction of the ceRNA network. Based on the four mRNAs, including ACTC1, FAM129A, OSBPL10, and EPHA2, Jiang et al*.* constructed a ceRNA network as a biomarker for BC patients [40]. Moreover, another study suggested that six biomolecules (LINC01198, PTPRD-AS1, SEMA3D, has-miR-216a, EPHA5, and DCLK1) from the ceRNA regulation network were significantly related to the excellent survival prognosis of BC, indicating that these characteristic molecules were high-risk factors for BC [41]. These discrepancies between previous studies and the present study probably attribute to different research methods and targets.

ADAMTS9-AS1 is an antisense lncRNA and has been reported in a variety of cancers. Fan et al*.* [42] reported that ADAMTS9-AS1 could serve as an independent prognostic marker for predicting the survival of patients with breast cancer. Another study by Xing and colleagues also indicated that ADAMTS9-AS1 could play a role in predicting the overall survival of patients with colon adenocarcinoma [43]. In our study, low expression of ADAMTS9-AS1 was associated with poor survival of BC patients, indicating that it had a significant prognostic value in BC.

Finally, we evaluated the predictive value of the three-lncRNA signature in BC. The resulted show that the risk score constructed based on the expression of these three lncRNAs was significantly related to overall survival in BC patients (*P*<0.05). Patients with high-risk scores faced an unfortunate tendency of worse overall survival, indicating that the three-lncRNA signature was a potential independent prognostic factor of BC.

## Conclusions

To sum up, we have analyzed DEGs between BC and adjacent-normal tissues and then constructed a BC-related ceRNA network comprising 49 DE lncRNAs, 17 DE miRNAs, and 36 DE mRNAs based on the TCGA database. Three lncRNAs were identified to function as predictive biomarkers of BC based on the overall survival. Notably, a high-risk score was significantly associated with poor overall survival of BC patients. The present study provides novel insights into the lncRNA-mediated ceRNA network and the potential of lncRNAs to be candidate prognostic biomarkers in BC, which could help better understand the pathological changes and pathogenesis of BC and be useful for clinical studies in the future.

## Supplementary Material

Supplementary Tables S1-S3

## Data Availability

The origin RNA-seq data used in our study were all downloaded from the TCGA (https://portal.gdc.cancer.gov/). All data analyzed during this study are included in this published article and its supplementary information files.

## Abbreviations

BC: bladder cancer
ceRNA: competing endogenous RNA
DE RNA: differentially expressed RNA
HR: hazard ratio
lncRNA: long non-coding RNA
MAPK: mitogen-activated protein kinase
MF: molecular function

## References

[1] Zhan Y., Du L., Wang L., Jiang X., Zhang S., Li J., Yan K., Duan W., Zhao Y., Wang L. et al. (2018) Expression signatures of exosomal long non-coding RNAs in urine serve as novel non-invasive biomarkers for diagnosis and recurrence prediction of bladder cancer. Mol. Cancer 17, 142 30268126 10.1186/s12943-018-0893-y PMC6162963

[2] Gou L., Liu M., Xia J., Wan Q., Jiang Y., Sun S., Tang M., Zhou L., He T. and Zhang Y. (2018) BMP9 Promotes the Proliferation and Migration of Bladder Cancer Cells through Up-Regulating lncRNA UCA1. Int. J. Mol. Sci. 19, 1116 29642505 10.3390/ijms19041116 PMC5979556

[3] Koufopoulou M., Miranda P.A.P., Kazmierska P., Deshpande S. and Gaitonde P. (2020) Clinical evidence for the first-line treatment of advanced urothelial carcinoma: Current paradigms and emerging treatment options. Cancer Treat. Rev. 89, 102072 32769039 10.1016/j.ctrv.2020.102072

[4] Rijnders M., de Wit R., Boormans J.L., Lolkema M.P.J. and van der Veldt A.A.M. (2017) Systematic Review of Immune Checkpoint Inhibition in Urological Cancers. Eur. Urol. 72, 411–423 28645491 10.1016/j.eururo.2017.06.012

[5] Ulitsky I. (2016) Evolution to the rescue: using comparative genomics to understand long non-coding RNAs. Nat. Rev. Genet. 17, 601–614 27573374 10.1038/nrg.2016.85

[6] Mercer T.R., Dinger M.E. and Mattick J.S. (2009) Long non-coding RNAs: insights into functions. Nat. Rev. Genet. 10, 155–159 10.1038/nrg2521 19188922

[7] Yao R.W., Wang Y. and Chen L.L. (2019) Cellular functions of long noncoding RNAs. Nat. Cell Biol. 21, 542–551 10.1038/s41556-019-0311-8 31048766

[8] Gong C., Li Z., Ramanujan K., Clay I., Zhang Y., Lemire-Brachat S. and Glass D.J. (2015) A long non-coding RNA, LncMyoD, regulates skeletal muscle differentiation by blocking IMP2-mediated mRNA translation. Dev. Cell 34, 181–191 10.1016/j.devcel.2015.05.009 26143994

[9] Xiong G., Liu C., Yang G., Feng M., Xu J., Zhao F., You L., Zhou L., Zheng L., Hu Y. et al. (2019) Long noncoding RNA GSTM3TV2 upregulates LAT2 and OLR1 by competitively sponging let-7 to promote gemcitabine resistance in pancreatic cancer. J. Hematol. Oncol. 12, 97 10.1186/s13045-019-0777-7 31514732 PMC6739963

[10] Wang A., Bao Y., Wu Z., Zhao T., Wang D., Shi J., Liu B., Sun S., Yang F., Wang L. and Qu L. (2019) Long noncoding RNA EGFR-AS1 promotes cell growth and metastasis via affecting HuR mediated mRNA stability of EGFR in renal cancer. Cell Death Dis. 10, 154 10.1038/s41419-019-1331-9 30770799 PMC6377662

[11] Chen M., Fan L., Zhang S.M., Li Y., Chen P., Peng X., Liu D.B., Ma C., Zhang W.J., Zou Z.W. and Li P.D. (2019) LINC01939 inhibits the metastasis of gastric cancer by acting as a molecular sponge of miR-17-5p to regulate EGR2 expression. Cell Death Dis. 10, 70 10.1038/s41419-019-1344-4 30683847 PMC6347617

[12] Zhan Y., Lin J., Liu Y., Chen M., Chen X., Zhuang C., Liu L., Xu W., Chen Z., He A. et al. (2016) Up-regulation of long non-coding RNA PANDAR is associated with poor prognosis and promotes tumorigenesis in bladder cancer. J. Exp. Clin. Cancer Res. 35, 83 10.1186/s13046-016-0354-7 27206339 PMC4873988

[13] Cao X., Xu J. and Yue D. (2018) LncRNA-SNHG16 predicts poor prognosis and promotes tumor proliferation through epigenetically silencing p21 in bladder cancer. Cancer Gene Ther. 25, 10–17 10.1038/s41417-017-0006-x 29234154

[14] Terracciano D., Ferro M., Terreri S., Lucarelli G., D'Elia C., Musi G., de Cobelli O., Mirone V. and Cimmino A. (2017) Urinary long noncoding RNAs in nonmuscle-invasive bladder cancer: new architects in cancer prognostic biomarkers. Transl. Res. 184, 108–117 10.1016/j.trsl.2017.03.005 28438520

[15] Salmena L., Poliseno L., Tay Y., Kats L. and Pandolfi P.P. (2011) A ceRNA hypothesis: the Rosetta Stone of a hidden RNA language? Cell 146, 353–358 10.1016/j.cell.2011.07.014 21802130 PMC3235919

[16] Huang C., Liao X., Jin H., Xie F., Zheng F., Li J., Zhou C., Jiang G., Wu X.R. and Huang C. (2019) MEG3, as a Competing Endogenous RNA, Binds with miR-27a to Promote PHLPP2 Protein Translation and Impairs Bladder Cancer Invasion. Mol. Ther. Nucleic Acids 16, 51–62 10.1016/j.omtn.2019.01.014 30826633 PMC6396102

[17] Shen L., Wang Q., Liu R., Chen Z., Zhang X., Zhou P. and Wang Z. (2018) LncRNA lnc-RI regulates homologous recombination repair of DNA double-strand breaks by stabilizing RAD51 mRNA as a competitive endogenous RNA. Nucleic Acids Res. 46, 717–729 10.1093/nar/gkx1224 29216366 PMC5778505

[18] Zhu J., Gu W. and Yu C. (2020) MATN1-AS1 promotes glioma progression by functioning as ceRNA of miR-200b/c/429 to regulate CHD1 expression. Cell Prolif. 53, e12700 10.1111/cpr.12700 31667976 PMC6985690

[19] Denny P., Feuermann M., Hill D.P., Lovering R.C., Plun-Favreau H. and Roncaglia P. (2018) Exploring autophagy with Gene Ontology. Autophagy 14, 419–436 10.1080/15548627.2017.1415189 29455577 PMC5915032

[20] Lin C.M. and Feng W. (2012) Microarray and synchronization of neuronal differentiation with pathway changes in the Kyoto Encyclopedia of Genes and Genomes (KEGG) databank in nerve growth factor-treated PC12 cells. Curr. Neurovasc. Res. 9, 222–229 10.2174/156720212801618983 22697417

[21] Sur S., Steele R., Shi X. and Ray R.B. (2019) miRNA-29b Inhibits Prostate Tumor Growth and Induces Apoptosis by Increasing Bim Expression. Cells 8, 1455 10.3390/cells8111455 31752117 PMC6912792

[22] Sun L., Fang Y., Wang X., Han Y., Du F., Li C., Hu H., Liu H., Liu Q., Wang J. et al. (2019) miR-302a Inhibits Metastasis and Cetuximab Resistance in Colorectal Cancer by Targeting NFIB and CD44. Theranostics 9, 8409–8425 10.7150/thno.36605 31754405 PMC6857048

[23] Yap T.A., Omlin A. and de Bono J.S. (2013) Development of therapeutic combinations targeting major cancer signaling pathways. J. Clin. Oncol. 31, 1592–1605 10.1200/JCO.2011.37.6418 23509311

[24] Braicu C., Buse M., Busuioc C., Drula R., Gulei D., Raduly L., Rusu A., Irimie A., Atanasov A.G., Slaby O. et al. (2019) A Comprehensive Review on MAPK: A Promising Therapeutic Target in Cancer. Cancers (Basel) 11, 10.3390/cancers11101618 PMC6827047 31652660

[25] Hoxhaj G. and Manning B.D. (2020) The PI3K-AKT network at the interface of oncogenic signalling and cancer metabolism. Nat. Rev. Cancer 20, 74–88 10.1038/s41568-019-0216-7 31686003 PMC7314312

[26] Bray F., Ferlay J., Soerjomataram I., Siegel R.L., Torre L.A. and Jemal A. (2018) Global cancer statistics 2018: GLOBOCAN estimates of incidence and mortality worldwide for 36 cancers in 185 countries. CA Cancer J. Clin. 68, 394–424 10.3322/caac.21492 30207593

[27] Tan W.S., Tan W.P., Tan M.Y., Khetrapal P., Dong L., deWinter P., Feber A. and Kelly J.D. (2018) Novel urinary biomarkers for the detection of bladder cancer: A systematic review. Cancer Treat. Rev. 69, 39–52 10.1016/j.ctrv.2018.05.012 29902678

[28] van Rhijn B.W., Burger M., Lotan Y., Solsona E., Stief C.G., Sylvester R.J., Witjes J.A. and Zlotta A.R. (2009) Recurrence and progression of disease in non-muscle-invasive bladder cancer: from epidemiology to treatment strategy. Eur. Urol. 56, 430–442 10.1016/j.eururo.2009.06.028 19576682

[29] Kamat A.M., Hahn N.M., Efstathiou J.A., Lerner S.P., Malmström P.-U., Choi W., Guo C.C., Lotan Y. and Kassouf W. (2016) Bladder cancer. Lancet North Am. Ed. 388, 2796–2810 10.1016/S0140-6736(16)30512-8 27345655

[30] Liu Y.H., Sun J., Yu J.Y., Ge W., Xiao X.G., Dai S.G. and Xiang Q.Y. (2019) LncRNA CACS15 accelerates the malignant progression of ovarian cancer through stimulating EZH2-induced inhibition of APC. Am. J. Transl. Res. 11, 6561–6568 31737207 PMC6834498

[31] Li X., Lv X., Li Z., Li C., Li X., Xiao J., Liu B., Yang H. and Zhang Y. (2019) Long Noncoding RNA ASLNC07322 Functions in VEGF-C Expression Regulated by Smad4 during Colon Cancer Metastasis. Mol. Ther. Nucleic Acids 18, 851–862 10.1016/j.omtn.2019.10.012 31739210 PMC6861657

[32] Kiuchi J., Komatsu S., Imamura T., Nishibeppu K., Shoda K., Arita T., Kosuga T., Konishi H., Shiozaki A., Okamoto K. et al. (2019) Low levels of tumour suppressor miR-655 in plasma contribute to lymphatic progression and poor outcomes in oesophageal squamous cell carcinoma. Mol. Cancer 18, 2 10.1186/s12943-018-0929-3 30609933 PMC6320607

[33] Fang J.H., Zhang Z.J., Shang L.R., Luo Y.W., Lin Y.F., Yuan Y. and Zhuang S.M. (2018) Hepatoma cell-secreted exosomal microRNA-103 increases vascular permeability and promotes metastasis by targeting junction proteins. Hepatology 68, 1459–1475 10.1002/hep.29920 29637568

[34] Miao L., Liu H.Y., Zhou C. and He X. (2019) LINC00612 enhances the proliferation and invasion ability of bladder cancer cells as ceRNA by sponging miR-590 to elevate expression of PHF14. J. Exp. Clin. Cancer Res. 38, 143 10.1186/s13046-019-1149-4 30940184 PMC6444615

[35] Gao R., Zhang N., Yang J., Zhu Y., Zhang Z., Wang J., Xu X., Li Z., Liu X., Li Z. et al. (2019) Long non-coding RNA ZEB1-AS1 regulates miR-200b/FSCN1 signaling and enhances migration and invasion induced by TGF-beta1 in bladder cancer cells. J. Exp. Clin. Cancer Res. 38, 111 10.1186/s13046-019-1102-6 30823924 PMC6397446

[36] Cheng Y., Zhang X., Li P., Yang C., Tang J., Deng X., Yang X., Tao J., Lu Q. and Li P. (2016) MiR-200c promotes bladder cancer cell migration and invasion by directly targeting RECK. Onco Targets Ther 9, 5091–5099 27574450 10.2147/OTT.S101067 PMC4993393

[37] Kumar B., Sinclair J., Khandrika L., Koul S., Wilson S. and Koul H.K. (2009) Differential effects of MAPKs signaling on the growth of invasive bladder cancer cells. Int. J. Oncol. 34, 1557–1564 19424573 10.3892/ijo_00000285

[38] Lin W., Xie J., Xu N., Huang L., Xu A., Li H., Li C., Gao Y., Watanabe M., Liu C. and Huang P. (2018) Glaucocalyxin A induces G2/M cell cycle arrest and apoptosis through the PI3K/Akt pathway in human bladder cancer cells. Int. J. Biol. Sci. 14, 418–426 10.7150/ijbs.23602 29725263 PMC5930474

[39] Xu Z., Wang C., Xiang X., Li J. and Huang J. (2019) Characterization of mRNA Expression and Endogenous RNA Profiles in Bladder Cancer Based on The Cancer Genome Atlas (TCGA) Database. Med. Sci. Monit. 25, 3041–3060 10.12659/MSM.915487 31020952 PMC6498884

[40] Jiang J., Bi Y., Liu X.P., Yu D., Yan X., Yao J., Liu T. and Li S. (2020) To construct a ceRNA regulatory network as prognostic biomarkers for bladder cancer. J. Cell. Mol. Med. 24, 5375–5386 10.1111/jcmm.15193 32233022 PMC7205833

[41] Sun Y., Zhu D., Xing H., Hou Y. and Liu Y. (2020) Screening of characteristic biomolecules related to bladder cancer based on construction of ceRNA regulation network. World J. Urol. 38, 2835–2847 10.1007/s00345-020-03086-2 32060632

[42] Fan C.N., Ma L. and Liu N. (2018) Systematic analysis of lncRNA-miRNA-mRNA competing endogenous RNA network identifies four-lncRNA signature as a prognostic biomarker for breast cancer. J. Transl. Med. 16, 264 10.1186/s12967-018-1640-2 30261893 PMC6161429

[43] Xing Y., Zhao Z., Zhu Y., Zhao L., Zhu A. and Piao D. (2018) Comprehensive analysis of differential expression profiles of mRNAs and lncRNAs and identification of a 14-lncRNA prognostic signature for patients with colon adenocarcinoma. Oncol. Rep. 39, 2365–2375 29565464 10.3892/or.2018.6324

