# Supplementary material for: Explore prognostic biomarker of bladder cancer based on competing endogenous network
Source: Biosci Rep. 2020 Dec 2;40(12):BSR20202463. doi: 10.1042/BSR20202463 (PMC7711062; doi:10.1042/BSR20202463)
Supplement: Supplementary Tables S1-S3 [file BSR-2020-2463_supp.pdf]

Supplementary Table 1 GO enrichment analysis of the DE mRNAs in the ceRNA network

Category	Term	Description	Count	*p* value
MF	GO:0001227	transcriptional repressor activity, RNA polymerase II transcription regulatory region sequence-specific binding	3	0.006
MF	GO:0003714	transcription corepressor activity	4	0.007
MF	GO:0042803	protein homodimerization activity	6	0.013
MF	GO:0051018	protein kinase A binding	2	0.037
MF	GO:0008201	heparin binding	3	0.039
MF	GO:0003682	chromatin binding	4	0.040
CC	GO:0005615	extracellular space	7	0.041
BP	GO:0000122	negative regulation of transcription from RNA polymerase II promoter	8	0.001
BP	GO:0001525	angiogenesis	4	0.010
BP	GO:0060070	canonical Wnt signaling pathway	3	0.012
BP	GO:0045944	positive regulation of transcription from RNA polymerase II promoter	7	0.013
BP	GO:2000144	positive regulation of DNA-templated transcription, initiation	2	0.014
BP	GO:0045892	negative regulation of transcription, DNA-templated	5	0.018
BP	GO:0090263	positive regulation of canonical Wnt signaling pathway	3	0.024
BP	GO:0043524	negative regulation of neuron apoptotic process	3	0.029
BP	GO:0006351	transcription, DNA-templated	9	0.038
BP	GO:0060045	positive regulation of cardiac muscle cell proliferation	2	0.044

BP	GO:0008285	negative regulation of cell proliferation	4	0.045
BP	GO:0002040	sprouting angiogenesis	2	0.049

GO, Gene Ontology; DE, Differently expressed; ceRNA, competing endogenous RNA; MF, molecular function; CC, cellular component; BP, biological process.

Supplementary Table 2 KEGG enrichment analysis of the DE mRNAs in the ceRNA network

Term	Description	Genes involved	Count	*p* value
hsa05206	MicroRNAs in cancer	RECK, CCNE1, ZFPM2, ZEB1, THBS1	5	0.006
hsa05200	Pathways in cancer	CCNE1, FGF9, JUN, MITF, WNT7A	5	0.017
hsa04010	MAPK signaling pathway	BDNF, DUSP2, FGF9, JUN	4	0.027
hsa04151	PI3K-Akt signaling pathway	CCNE1, FGF9, PRKAA2, THBS1	4	0.048

KEGG, Kyoto Encyclopedia of Genes and Genomes; DE, Differently expressed; ceRNA, competing endogenous RNA; MAPK, mitogen-activated protein kinase signaling pathway; PI3K-Akt, phosphatidylinositol-3 kinase-protein kinase B.

Supplementary Table 3 The correlation between the three lncRNA expression and clinicopathological characteristics of BC patients

Variables	AC112721.1			TMPRSS11GP			ADAMTS9-AS1		
	Low	High	*p*	Low	High	*p*	Low	High	*p*
Age									
>60	141	152	0.214	156	137	0.032	128	165	<0.001
≤60	59	48		44	63		72	35	
Gender									
Female	51	54	0.733	49	56	0.426	48	57	0.306
Male	149	146		151	144		152	143	
Stage									
I & II	95	33	<0.001	72	56	0.086	88	40	<0.001
III & IV	105	167		128	144		112	160	
Grade									
Low	18	3	0.001	15	6	0.044	15	6	0.044
High	182	197		185	194		185	194	
Lymph node									
Yes	43	85	<0.001	57	71	0.097	46	82	0.003
No	128	103		124	107		121	110	
Unknown	29	12		19	22		33	8	
